# A reconstructed database of historic bluefin tuna captures in the Gibraltar Strait and Western Mediterranean

**DOI:** 10.1016/j.dib.2017.11.028

**Published:** 2017-11-16

**Authors:** Josué M. Polanco-Martínez, Ángela M. Caballero-Alfonso, Unai Ganzedo, José J. Castro-Hernández

**Affiliations:** a Basque Centre for Climate Change (BC3), 48940 Leioa, Spain; b UMR CNRS 5805 EPOC, Université de Bordeaux, 33615 Pessac, France; c Otago Museum, 419 Great King Street, PO Box 6202, Dunedin 9059, New Zealand; d DigitalGlobe, Inc., 2325 Dulles Corner Blvd, Suite 1000, Herndon, VA 20171, USA; e University of Las Palmas de Gran Canaria, I.U. EcoAqua, Campus U. de Tafira, Edificio de Ciencias Básicas, Fac. Ciencias del Mar, sn., 35017 Las Palmas de Gran Canaria, Spain

**Keywords:** Data reconstructions, DINEOF, Missing data, Historic Bluefin tuna captures, Gibraltar Strait and Western Mediterranean

## Abstract

This data paper presents a reconstruction of a compilation of a small but consistent database of historical capture records of bluefin tuna (Thunnus thynnus; BFT hereafter) from the Gibraltar Strait and Western Mediterranean (Portugal, Spain and Italy). The compilation come from diverse historical and documentary sources and span the time interval from 1525 to 1936 covering a period of 412 years. There is a total of 3074 datum, which reach up to 67.83% of the total implying a 32.17% of missing data. However, we have only reconstructed the captures for the time interval 1700–1936 and we provide these reconstructions only for this time interval and for 9 out of 11 series due to the scarcity and inhomogeneity of the two oldest capture time series. This reconstructed database provides an invaluable opportunity for fisheries and marine research as well as for multidisciplinary research in climate change.

**Specifications Table**

**Table:** 

Subject area	Marine Sciences
More specific subject area	Fisheries research
Type of data	Text file, figure
How data was acquired	Statistical reconstruction of a compilation of historical BFT captures
Data format	Raw, reconstructed
Experimental factors	Not applicable
Experimental features	Statistical reconstructions by means of Data Interpolating Empirical Orthogonal Functions (DINEOF)
Data source location	The statistical reconstructions were fed by historical captures from the followings traps:
	Barril (Portugal): N36° 42' 00", W7° 47' 59"
	Medo das Casas (Portugal): N36° 53' 59", W7° 12' 00"
	Saline (Sardinia, Italy): N41° 00' 00", E8° 00' 00"
	Porto Paglia (Sardinia, Italy): N39° 45' 00", E8° 05' 59"
	Porto Scuso (Sardinia, Italy): N39° 19' 48", E8° 05' 59"
	Isola Piana (Sardinia, Italy): N38° 42' 00", E8° 24' 00"
	Bonagia (Sicily, Italy): N38° 30' 00", E12° 35' 59"
	San Giuliano (Sicily, Italy):N38° 00' 00", E12° 05' 59"
	Formica (Sicily, Italy): N37° 30' 00", E12° 11' 59"
Data accessibility	Data is with this article
Related research article	Ganzedo, U., Polanco-Martínez, J. M., Caballero-Alfonso, Á. M., Faria, S. H., Li, J., & Castro-Hernández, J. J. (2016). Climate effects on historic bluefin tuna captures in the Gibraltar Strait and Western Mediterranean. Journal of Marine Systems, 158:84–92.

**Value of the Data**

• The reconstructed historical capture records could be useful to analyze the long-term fluctuations of bluefin tuna from the Gibraltar Strait and Western Mediterranean.
• This database provides an invaluable opportunity for fisheries and marine research (e.g., resources management) as well as for multidisciplinary research in climate change.
• This datasets will be beneficial to understand the bluefin tune population dynamics and their relationship with different environmental variables

## 1 Data

**Fig. 1:**
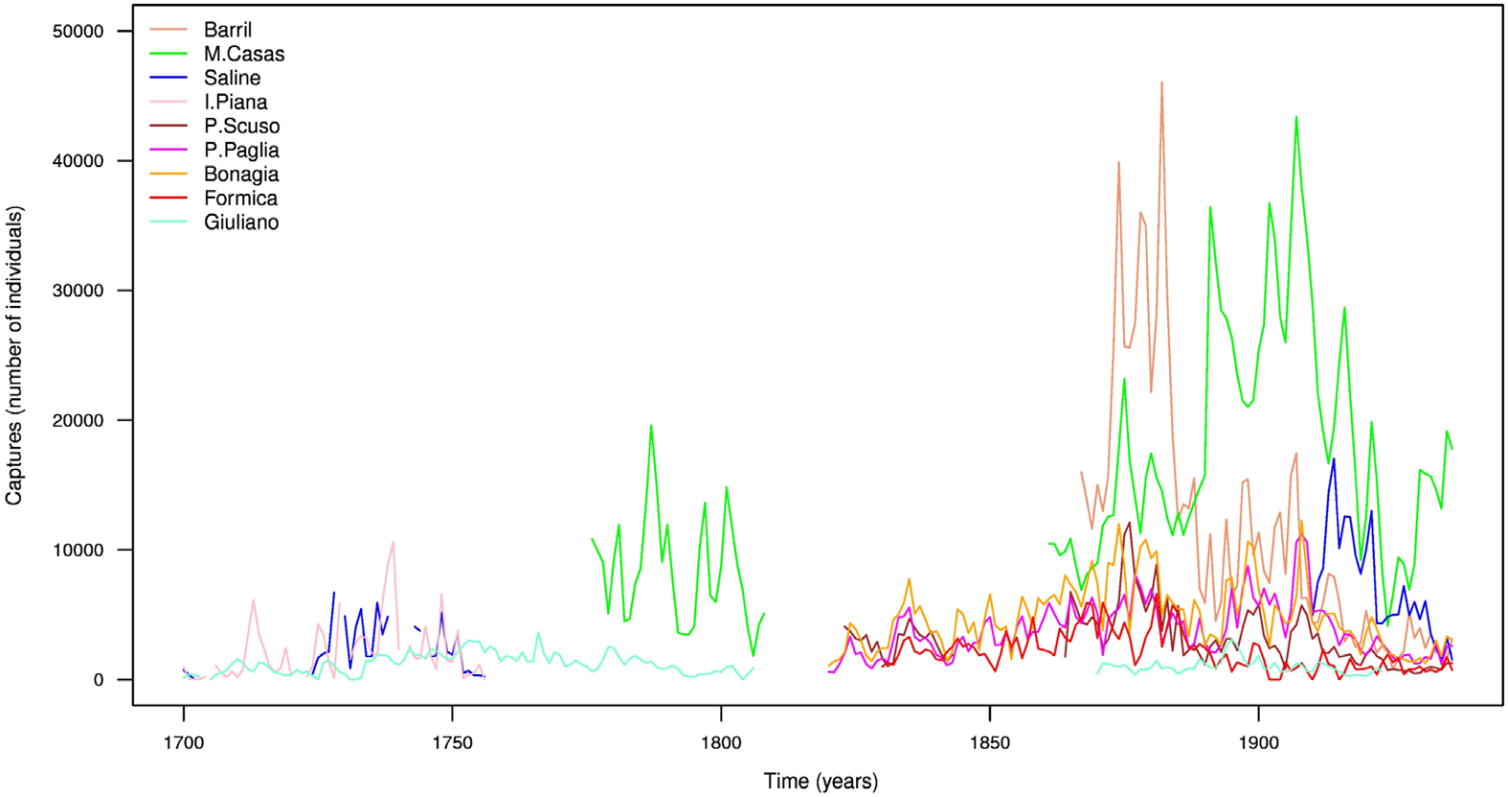
Time series of the BFT captures (number of individuals or tunas) for each trap for the time interval 1700–1936.

**Fig. 2:**
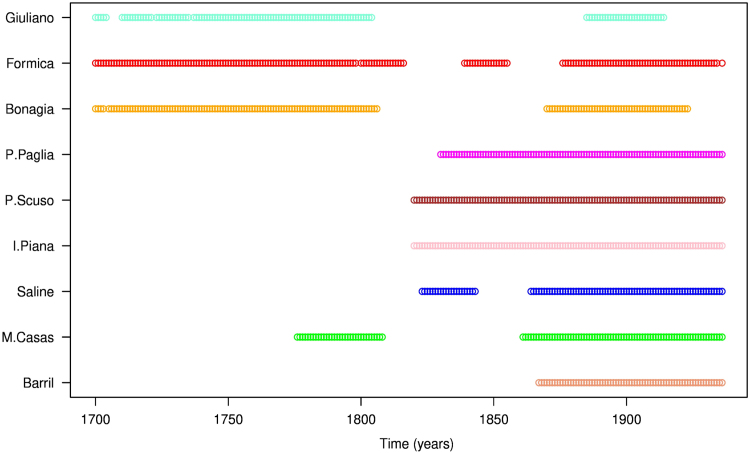
Presence (o) and absence (blank) and the number of elements (*N*) of the BFT captures time series by each tuna-trap for the time interval 1700–1936.

**Fig. 3:**
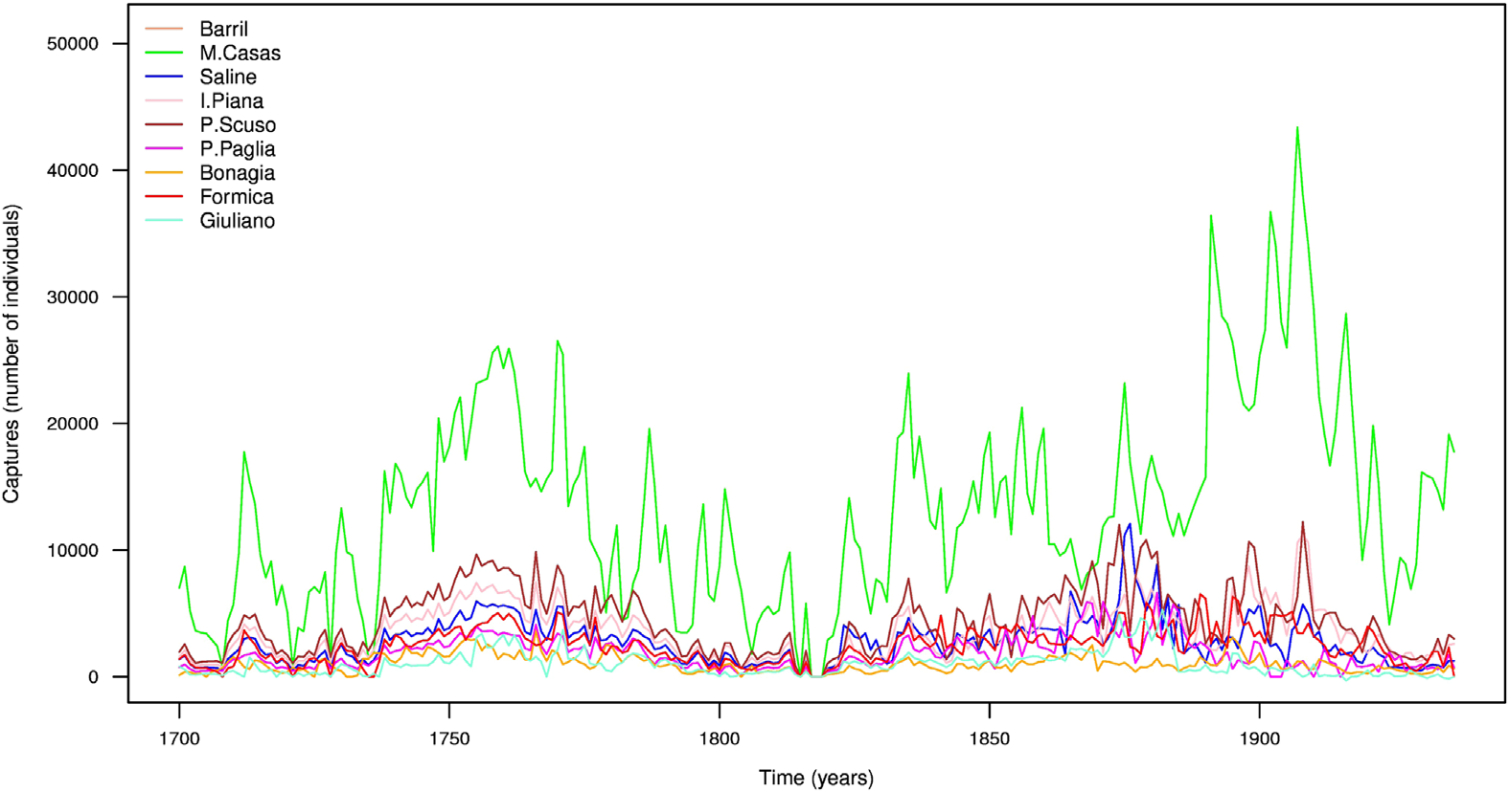
BFT captures reconstructed via DINEOF for the time interval 1700–1936.

The historical BFT captures span the time interval from 1525 to 1936 covering a period of 412 years (Fig. 1). There is a total of 3074 datum, which reach up to 67.83% of the total implying a 32.17% of missing data (Fig. 2). Data were manually digitalized from diverse documentary and historical sources as well as some “recent” publications [1], [2], [3], [4], [5], [6], [7], [8], [9]. Moreover, the database was double-checked due to potential typographical errors by the investigators. In addition, we have compared visually and quantitatively (as much as possible) our compilations with other previous works [2], [4], [6], [7], [8], [9], [10]. After a preliminary inspection, we decided to limit our data reconstructions to the time interval from 1700 to 1936, due to the scarcity and inhomogeneity of the two oldest capture time series (Conil and Zahara; Fig. 1 in [9]). As a consequence of these drawbacks, we removed Conil and Zahara in our data reconstructions (Fig. 3).

## 2 Experimental design, materials and methods

We have reconstructed the missing data using the Data INterpolating Empirical Orthogonal Functions technique (DINEOF) [11], [12], [13]; as implemented in the R package sinkr [14]. This statistical technique of data reconstruction is based on the decomposition of the time series into Empirical Orthogonal Functions (EOF), and it was first applied to fisheries by [13]. DINEOF is a self-consistent method for reconstructions missing values contained in geophysical data (i.e., oceanographic, meteorological, etc.) [15]. This statistical method is based on the fact that an optimal number of EOFs, usually very small if compared to the total number of EOFs, retains a large fraction of the total variance of the whole dataset. The DINEOF method fills the missing data by means of an iterative process [12], [13]: 1) the leading EOF is computed; 2) the leading EOF is used to estimate the anomalies at the missing points; 3) the process is iterated until convergence in the anomalies at the missing values is achieved from one iteration to the next within a prescribed tolerance level; 4) once the convergence is reached, the number of computed EOFs increases, from 1 to 2 and next to k_max_ EOFs; and 5) there is an estimate for the missing data reconstructed after convergence is achieved with a reconstruction computed using 1, 2, …, k_max_ EOFs. The optimum number of EOFs to be used in the reconstructions is defined by means of the cross-validation technique [16]. However, in this data paper, we have used the maximum number of EOFs, which corresponds to the number of reconstructed time series (i.e., 9 series).

## References

[1] Sarmiento F.M. (1757). De los atunes y sus transmigraciones y conjeturas sobre la decadencia de las almadrabas y sobre los medios para restituirlas.

[2] López-Capont F. (1997). La etapa pesquera del padre Sarmiento y su época: de los atunes, y sus transmigraciones y conjeturas, sobre la decadencia de las almadrabas, sobre los medios para restituirlas.

[3] Ravier C., Fromentin J.M. (2001). Long-term fluctuations in the eastern Atlantic and Mediterranean bluefin tuna population. ICES J. Mar. Sci..

[4] Ravier C. (2003). Fluctuations à long terme du thon rouge – validité, origines et conséquences (PhD Thesis).

[5] Lemos R.T., Gomes J.J. (2004). Do local environmental factors induce daily and yearly variability in bluefin tuna (Thunnus thynnus) trap catches?. Ecol. Model..

[6] Ganzedo U. (2005). Efecto de las variaciones climáticas en la distribución espacio-temporal de Thunnus thynnus thynnus (Linnaeus 1758) y Thunnus alalunga (Bonnaterre 1788) en el Océano Atlántico (PhD Thesis).

[7] Ganzedo U., Zorita E., Solari A.P., Chust G., Del Pino A.S., Polanco J., Castro J.J. (2009). What drove tuna catches between 1525 and 1756 in southern Europe?. ICES J. Mar. Sci..

[8] Caballero-Alfonso A.M. (2011). Recent and historical climate variability effects on the population dynamics of several marine species (PhD Thesis).

[9] Ganzedo U., Polanco-Martínez J.M., Caballero-Alfonso A.M., Faria S.H., Li J., Castro-Hernández J.J. (2016). Climate effects on historic bluefin tuna captures in the Gibraltar Strait and Western Mediterranean. J. Mar. Syst..

[10] Polanco-Martínez J.M. (2012). Aplicación de técnicas estadísticas en el estudio de fenómenos ambientales y ecosistémicos (PhD thesis). https://addi.ehu.es/handle/10810/11295.

[11] Alvera-Azcárate A., Barth A., Rixen M., Beckers J.M. (2005). Reconstruction of incomplete oceanographic data sets using empirical orthogonal functions: application to the Adriatic Sea surface temperature. Ocean Model..

[12] Ganzedo U., Alvera-Azcarate A., Esnaola G., Ezcurra A., Saenz J. (2011). Reconstruction of sea surface temperature by means of DINEOF: a case study during the fishing season in the Bay of Biscay. Int. J. Remote Sens..

[13] Ganzedo U., Erdaide O., Trujillo-Santana A., Alvera-Azcárate A., Castro J.J. (2013). Reconstruction of spatiotemporal capture data by means of orthogonal functions: the case of skipjack tuna (Katsuwonus pelamis) in the central-east Atlantic. Sci. Mar..

[14] M. Taylor, A collection of functions featured on the blog 'me nugget'; R package ver. 1.0, 2004, 〈https://github.com/menugget/sinkr〉 (Accessed 31 July 2017).

[15] Beckers J., Rixen M. (2003). EOF calculations and data filling from incomplete oceanographic data sets. J. Atmos. Ocean. Tech..

[16] Wilks D. (1995). Statistical Methods in the Atmospheric Science.

