# Supplementary material for: A reconstructed database of historic bluefin tuna captures in the Gibraltar Strait and Western Mediterranean
Source: Data Brief. 2017 Nov 16;16:206–10. doi: 10.1016/j.dib.2017.11.028 (PMC5712807; doi:10.1016/j.dib.2017.11.028)
Supplement: Supplementary file 1 — Supplementary material [file mmc1.pdf]

October 18, 2017
Bordeaux, France

Reference: Manuscript "**A reconstructed database of historic bluefin tuna captures in the Gibraltar Strait and Western Mediterranean**" by Josué M. Polanco-Martínez*, Ángela M. Caballero-Alfonso, Unai Ganzedo and José J. Castro-Hernández, submitted for consideration as an *data paper* in ***Data in Brief (DiB)***.

There are no conflicts of interest associated with this work. All correspondence should be sent to Josué M. Polanco-Martínez. (josue.m.polanco@gmail.com / josue.polanco@bc3research.org).

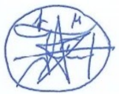

Sincerely, Dr. Josué M. Polanco Martínez on behalf of all co-authors of the manuscript.

UMR CNRS 5805 EPOC, Université de Bordeaux, 33615 Pessac, France.

Basque Centre for Climate Change (BC3), 48940 Leioa, Spain.
